# Supplementary material for: Rapid vessel segmentation and reconstruction of head and neck angiograms using 3D convolutional neural network
Source: Nat Commun. 2020 Sep 24;11:4829. doi: 10.1038/s41467-020-18606-2 (PMC7518426; doi:10.1038/s41467-020-18606-2)
Supplement: Supplementary file 2 — Supplementary Information [file 41467_2020_18606_MOESM2_ESM.pdf]

Rapid vessel segmentation and reconstruction of head and neck angiograms using 3D convolutional neural network

Supplementary Table 1. Comparison of image quality between CerebralDoc and Manual processing

	CerebralDoc (Percentiles, P_{25}, P_{75})	Manual processing (Percentiles, P_{25}, P_{75})	*P* value
VR	(3,3)	(2,3)	<0.001
MIP	(3,3)	(2,2)	<0.001
MPR/CPR	(3,3)	(3,3)	0.617

Mann–Whitney test were used for comparing two groups.

Supplementary Table 2. Comparison with previous work, two categories of methods (rule-based and DL-based) are listed.

Type	Study	Test set patients	Image	AO	Carotid artery	Cerebral vessel
				DSC	DSC	DSC
Rule-based	Olivier Cuisenaire et al. (2009)	28	CTA	-	90.3	-
	Danilo Babin et al. (2013)	4	CTA	-	-	0.82
	Duan et al. (2016)	10	CTA	0.93	-	-
DL	Michelle Livne et al. (2019)	66	MRA	-	-	0.88
	Ours	1826	CTA	0.975		
