# Supplementary material for: Rapid vessel segmentation and reconstruction of head and neck angiograms using 3D convolutional neural network
Source: Nat Commun. 2020 Sep 24;11:4829. doi: 10.1038/s41467-020-18606-2 (PMC7518426; doi:10.1038/s41467-020-18606-2)
Supplement: Supplementary file 3 — Supplementary Software [file 41467_2020_18606_MOESM3_ESM.docx]

**1. System requirements**

- All software dependencies and operating systems (including version numbers)

Chrome browser (version 83.0.4103.61, optional) and open in a network environment.

- Versions the software has been tested on

Version online_1.6.4/1.6.7/1.6.5.

- Any required non-standard hardware

None.

**2. Installation guide**

- Instructions

The software is a web version, users only need to click on the link to open on the chrome web page, and then use the account password to login.

- Typical install time on a "normal" desktop computer

No installation required.

**3. Demo and Instructions for use**

CerebralDoc is an AI-powered clinical post-processing system for head and neck CT angiography. It automatedly pulls raw data from workstation through network connection. All relevant medical image data is processed and presented in this workstation.

- Instructions to run on data

First, click the link <http://test.platform.shukun.net/login> to enter the login screen.

User name: test; Passwords: 123456


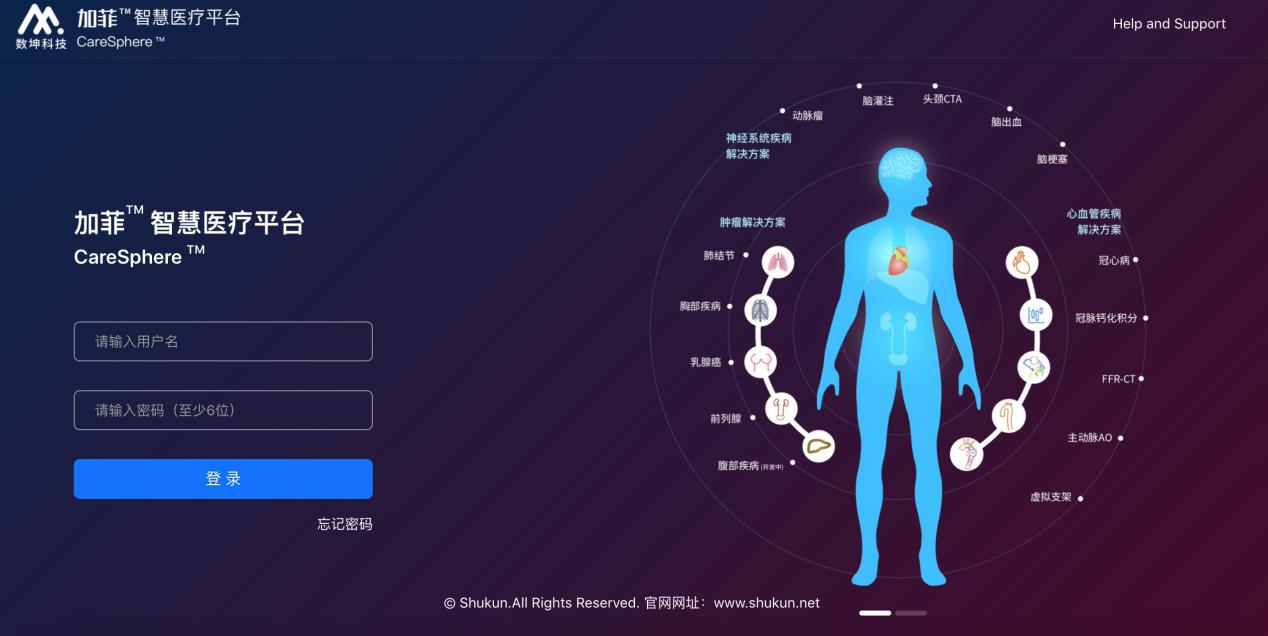


Login

Pass words

User name

After logging in, you should click the button “Global Site – English” firstly for suitable language, then click the “Digital Brain” and “CerebralDoc”, so that you can see the demo cases processed by CerebralDoc. These demo data contain a total of 164 clinical routine cases of head and neck CTA scan examination. In order to ensure information security, we have anonymized all calculated cases.


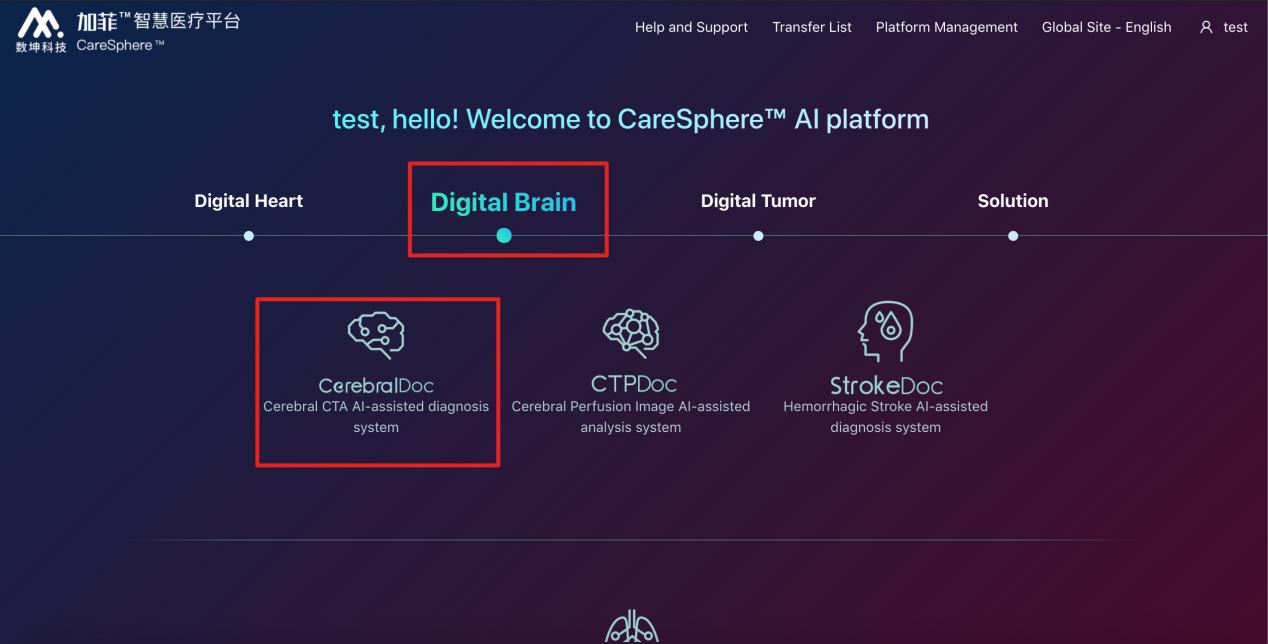


It requires attention that the “Exam Time” needs to select a longer time range (over 6 months) to acquire whole cases list. After that, click on a case to enter the post-process and diagnosis module.


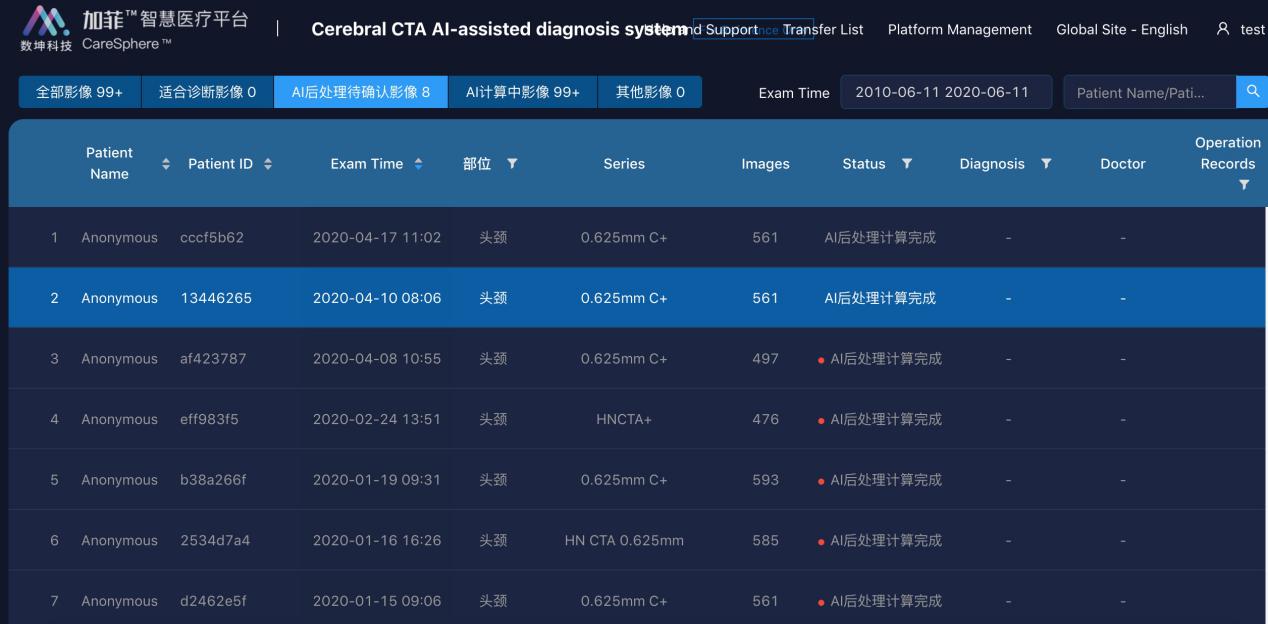


- Expected output

On the left we can see the automatic arteries naming results of the system. And on the right, there are four sequences including VR, MIP, CPR, curved-MPR and source images generated by deep learning algorithm. These five different types of image sequences are interconnected and interactive. When you scroll the images, the segmentation parts on the source image will also have simultaneous corresponding changes. The whole process from post-processing is completely automated, requiring no manual intervention.


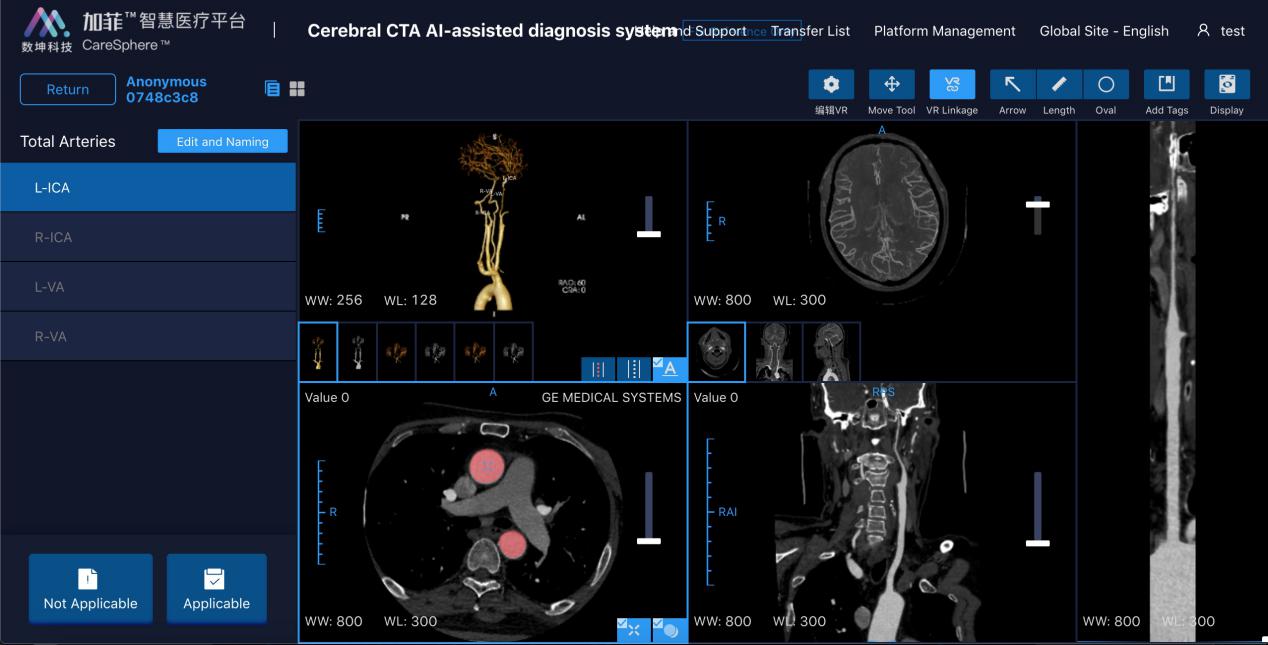


Vessel segmentation result

Source

CPR

MPR

MIP

VR

The system could automatedly mark the arteries segmentation results (red color) on the source image, and also can hide the vessel segmentation result by clicking the “Hide Segmentation（S）” marks.


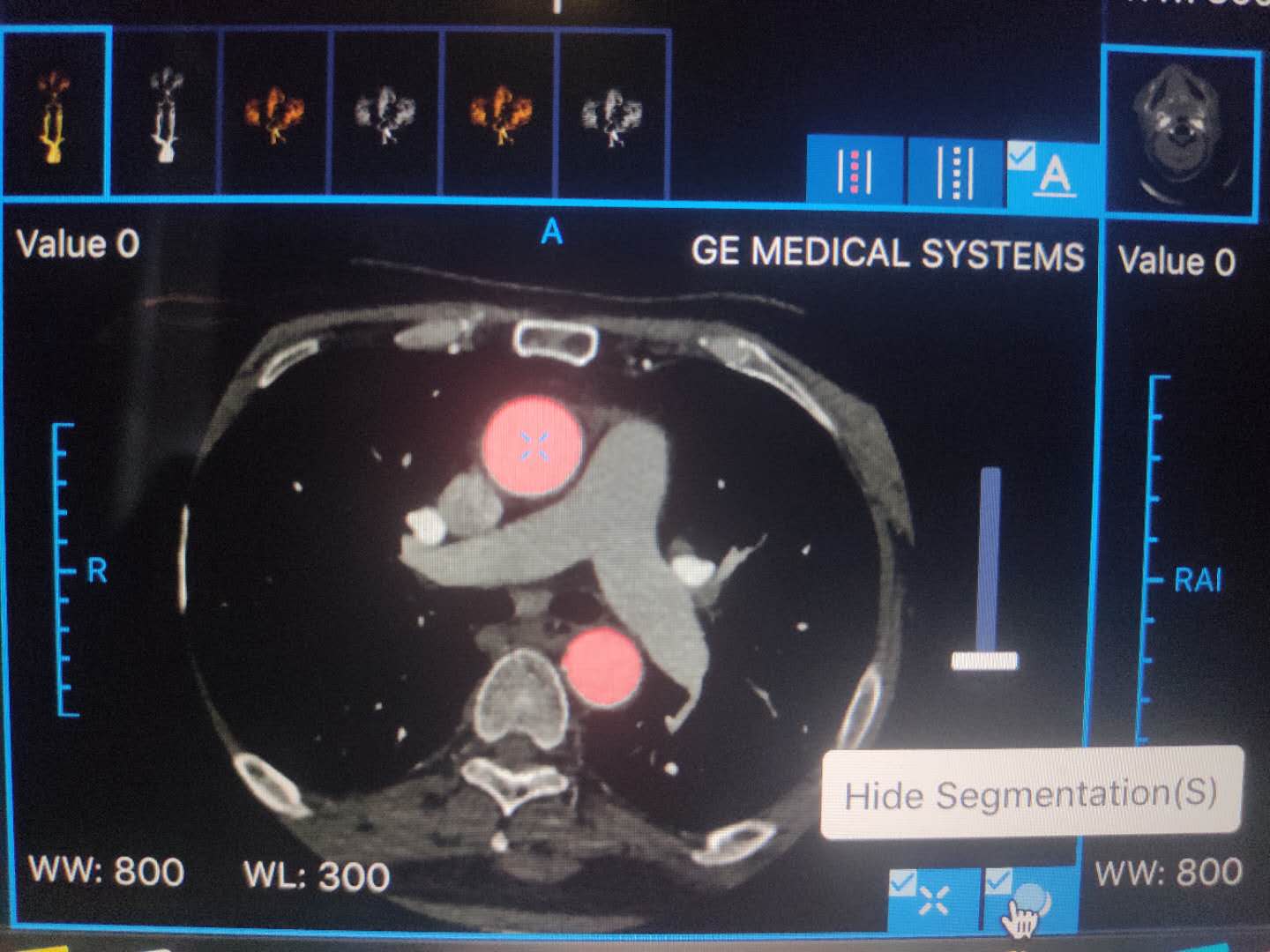


- Expected run time for demo on a "normal" desktop computer

All the data has been run automatically on the software; users can view the processing results in real time.
